# Optimization of competency in obstetrical emergencies: a role for simulation training

**DOI:** 10.1007/s00404-013-3111-6

**Published:** 2013-12-18

**Authors:** Cécile Monod, Cora A. Voekt, Martina Gisin, Stefan Gisin, Irene M. Hoesli

**Affiliations:** 1 Department of Obstetrics, University Hospital Basel, Spitalstrasse 21, 4031 Basel, Switzerland; 2 Department of Anaesthesiology, University Hospital Basel, Spitalstrasse 21, Basel, Switzerland; 3 Swiss Center for Medical Simulation “SimBa”, University Hospital Basel, Spitalstrasse 21, 4031 Basel, Switzerland

**Keywords:** Obstetrics, Simulation training, Team communication

## Abstract

**Purpose:**

In obstetrical emergency situations, optimal management requires the immediate coordinated actions of a multi-disciplinary and multi-professional team. This study investigated the influence of simulation training on four specific skills: self-confidence, handling of emergency situation, knowledge of algorithms and team communication.

**Methods:**

Clinical algorithms were first presented to the participants. Training for six emergency situations (shoulder dystocia, postpartum haemorrhage, pre-eclampsia, maternal basic life support, neonatal resuscitation and operative vaginal birth) was performed using high- and low-fidelity simulation mannequins. General impression of the simulation training and the four above-mentioned skills were evaluated anonymously through a self-assessment questionnaire with a five-point Likert scale immediately after the training and 3 months later.

**Results:**

From November 2010 to March 2012, 168 participants, distributed over six one-day courses, took part in the training. 156 participants returned the questionnaire directly after the course (92.9 %). The questionnaire return rate after 3 months was 36.3 %. The participants gave higher Likert scale answers for the questions on the four specific skills after 3 months compared to immediately after the course. The improvement was statistically significant (*p* ≤ 0.05) except for the question regarding team communication.

**Conclusion:**

Implementation of simulation training strengthens the professional competency.

## Introduction

Obstetrical emergencies induce intense stress and appropriate management of these situations requires immediate coordinated actions of a multi-disciplinary and multi-professional team.

Simulation training in obstetrics is a promising method for improving safety during birth for women and their newborns. The Confidential Enquiries Report into maternal deaths in the United Kingdom [1] and the Confidential Enquiry into Stillbirths and Deaths in Infancy (CESDI Report) [2] reported that 50 % of maternal deaths and 75 % of intrapartum foetal deaths could be avoided by optimal obstetrical management.

Several authors have shown evidence of efficacy of simulation training in obstetrical management. Reynolds et al. [3] observed an improvement in self-perceived knowledge and skills after participation in a 1-day simulation course for obstetrical emergencies and Draycott et al. [4, 5] described even a significant reduction of neonatal morbidity after introducing a compulsory training programme.

Nowadays, increased alertness is directed towards communication and team training as an essential component of optimal management of obstetrical emergencies [6–8].

The purpose of this study was to determine the influence of a one-day multi-disciplinary and multi-professional simulation training course for obstetrical emergencies based on the self-assessment of four specific skills: self-confidence, handling of the emergency situation, knowledge of algorithms and team communication.

We also analysed the influence of professional experience on self-perceived improvement of the four investigated specific skills 3 months after the training.

## Materials and methods

In this observational study, six multi-professional, multi-disciplinary obstetrical simulation training courses were organized between November 2010 and March 2012 by the Department of Obstetrics in collaboration with the Departments of Anaesthesiology and Neonatology at the University Hospital of Basel, Switzerland. The courses were conducted at the Swiss Center for Medical Simulation (“SimBa”), Basel, Switzerland. Midwifes and obstetricians from different public and private Swiss hospitals were allowed to participate in these courses. After a brief presentation of the clinical algorithms and essentials of medical simulation and crisis resource management (CRM), the participants trained in small groups and retained their normal function (junior or senior obstetrician, midwife) under supervision of multi-professional tutor-teams in six different obstetrical emergency situations: shoulder dystocia, postpartal haemorrhage, instrumental delivery for foetal distress, pre-eclampsia/eclampsia, maternal Basic Life Support and neonatal resuscitation. The participants worked on different simple and high-fidelity mannequins: Noelle^®^ (Gaumard, Miami, FL, USA) for shoulder dystocia training, Ambu^®^ Man (Ambu, Ballerup, Denmark) for maternal resuscitation, SimMan^®^ Classic and 3G (Laerdal Medical, Stavanger, Norway) for postpartal haemorrhage and pre-eclampsia/eclampsia and obstetrical leather models for operative vaginal delivery. To further augment the realism of the emergency situations and allow training of communication with the patient, scripted role players were acted as standardized patients at all the training stations.

The training groups of participants consisted of a maximum of six participants, namely, two midwives, two junior and two senior obstetricians. Three of them (one of each function) actively participated in the clinical scenario, while the remaining three were observants. With the consent of the participants, the training was videotaped at three of the six stations (pre-eclampsia/eclampsia, postpartal haemorrhage and shoulder dystocia). The tutors were comprised experienced obstetricians, anaesthesiologists and neonatologists with special education in leading simulation training courses. After each scenario, a debriefing was held to give immediate feedback to the participants and their performance. Where applicable, relevant extracts from the video-recordings were shown to strengthen the learning experience of the teams. The video records were erased after the course.

Immediately (questionnaire a) and 3 months after training (questionnaire b) the participants filled up an anonymous self-assessment questionnaire with a five-point Likert scale, evaluating subjective changes in the following skills: self-confidence, handling of the obstetrical emergency situation, knowledge of algorithms and team communication. The questions of our questionnaires a and b are not published in any other study. At the same time, the participants also answered a six-point Likert scale questionnaire on general acceptance of simulation training, a questionnaire previously used in a study by Blum et al. [9], together with the professional experience and function of each participant. The voluntary and anonymous questionnaires were a standard part of assessment of the institutional quality of service and, therefore, ethical approval was not necessary.

The first written questionnaire was filled up directly after the course and the second electronically in SurveyMonkey^®^ 3 months after the course.

### Statistical analysis

The answers from the self-assessment questionnaire directly and 3 months after the training were compared with Fisher’s exact test for count data. A *p* value of ≤0.05 was considered significant. Sample size was determined by the number of participants in the 3-year period.

Statistical analysis was performed using R, Version 2.12.0 [10].

## Results

A total of 168 participants took part in the six simulation training sessions. 156 participants returned the questionnaire regarding their self-perceived experience directly after the training (questionnaire a 92.9 %). 74 participants completed the electronical questionnaire on SurveyMonkey^®^ 3 months after the course (questionnaire b). The overall return rate of this questionnaire was 36.3 %. 153 participants provided information about their clinical function: 51 (33.3 %) midwives and 102 (66.7 %) obstetricians took part in the training. 156 participants indicated their level of professional experience: 40 (25.7 %) had 0–2 years, 30 (19.2 %) 2–5 years, 34 (21.8 %) 5–10 years and 52 (33.3 %) >10 years of professional experience. During the 3-month interval between the two questionnaires, many of the participants indicated that they had been confronted with at least one of the simulated emergencies from the course.

Directly after the course, the participants answered questions about the use of simulation training in medicine in general and about each of the six scenarios. Answers were indicated using a Likert scale, ranging from 1 (strongly disagree) to 6 (strongly agree). The participants considered simulation training for obstetrical emergencies as a useful method to train as a team for the management of emergency situations [5.61 (95 % CI: 5.49–5.74)] and to improve patient safety [5.71 (95 % CI: 5.60–5.83)]. When asked if they would have rather train alone, they strongly disagreed [Likert scale of 1.72 (95 % CI: 1.52–1.91)]. When asked if they felt exposed and observed, they disagreed [Likert scale of 3.91 (95 % CI: 3.66–4.15)]. Although we used patient actors in the training, the participants gave a mean score of 3.45 (95 % CI: 3.23–3.67) for the question regarding training the communication with the patient (I learned how to communicate with the patient).

**Fig. 1:**
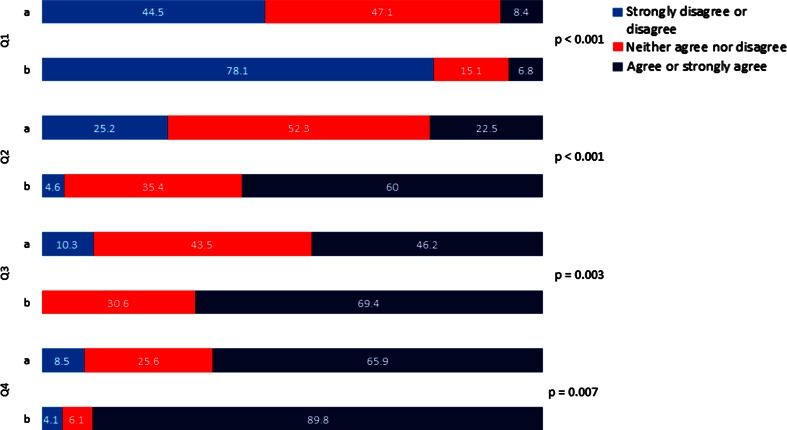
Overall Likert scale answers for Questions 1–4 directly and 3 months after the training. **a** Directly after the course. **b** 3 months after the course. *Question 1* I felt helpless. *Question 2* I felt I had the emergency situation under control. *Question 3* I had therapeutic algorithms in mind. *Question 4* **a** Directly after the course: during the scenarios I had the impression that the communication within the team improved. **b** 3 months after the course: my way of communicating in emergency situations improved

They gave a mean score of 4.94 (95 % CI: 4.8–5.09) for the question regarding training in communication and behaviour within the team (I learned how to communicate and how to behave in a team). The participants answered four questions about their feeling of self-perceived confidence, handling of emergency situations, remembering the clinical algorithms and the evolution of their ability to communicate within the team. For ease of analysis, we chose to combine the five-point Likert scale answers into three groups: strongly disagree or disagree, neither agree nor disagree, agree or strongly agree. In the overall analysis, there was statistically significant improvement in self-perceived competency 3 months after the course compared to directly after the course for the first three questions (*p* ≤ 0.05). There was a trend toward improvement in team communication competency 3 months after the course compared to directly after the course; however, this did not reach statistical significance (*p* = 0.07) (Fig. 1).

**Fig. 2:**
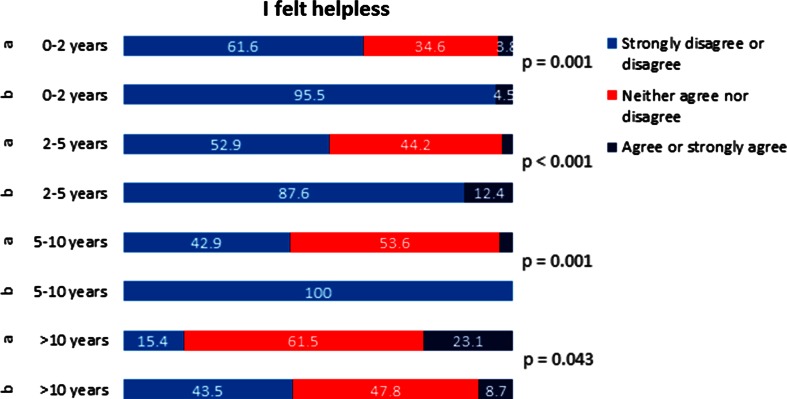
Likert scale answers for *Question 1* “I felt helpless”, plotted against professional experience of participants. **a** Directly after the course. **b** 3 months after the course

**Fig. 3:**
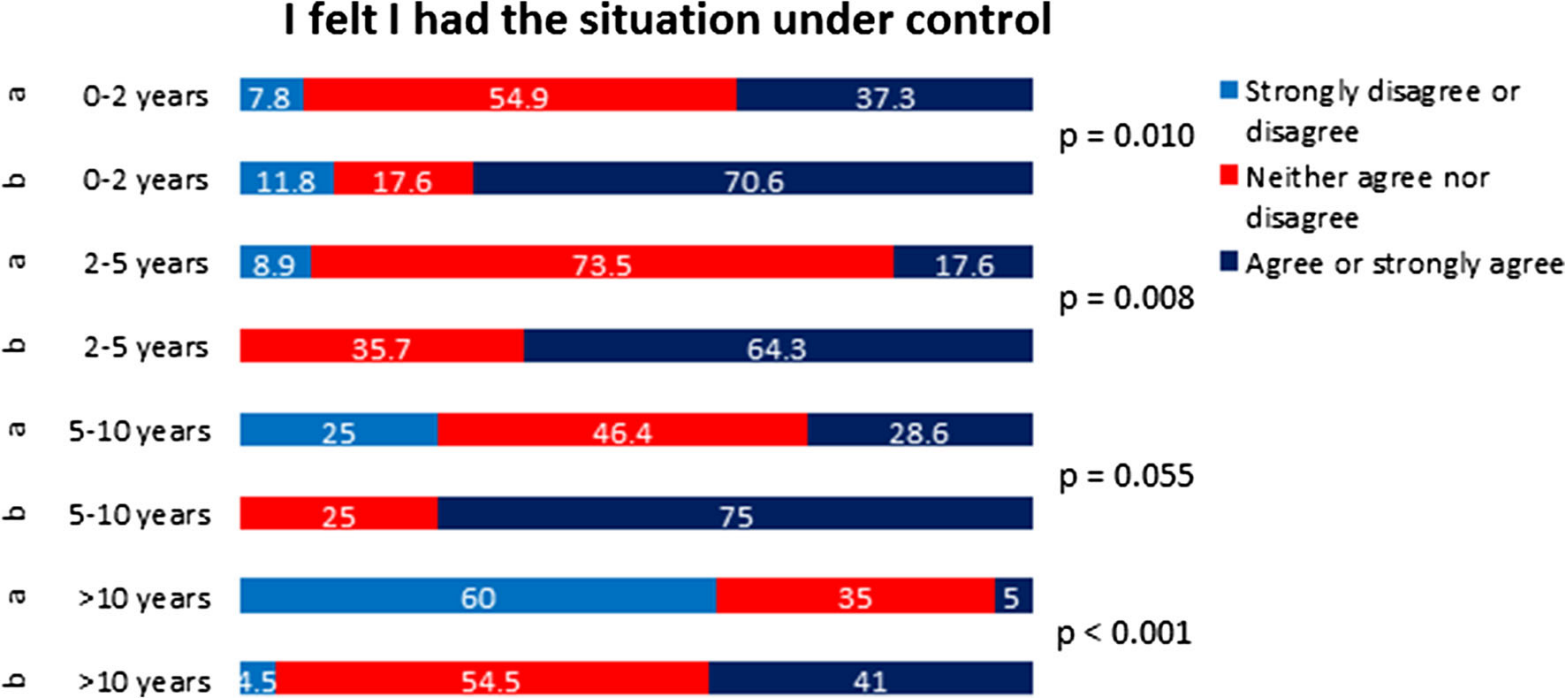
Likert scale answers for *Question 2* “I felt I had the emergency situation under control”, plotted against professional experience of participants. **a** Directly after the course. **b** 3 months after the course

**Fig. 4:**
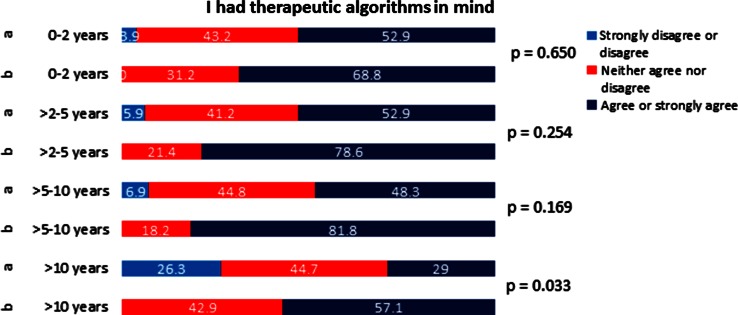
Likert scale answers for *Question 3* “I had the therapeutic algorithms in mind”, plotted against professional experience of participants. **a** Directly after the course. **b** 3 months after the course

**Fig. 5:**
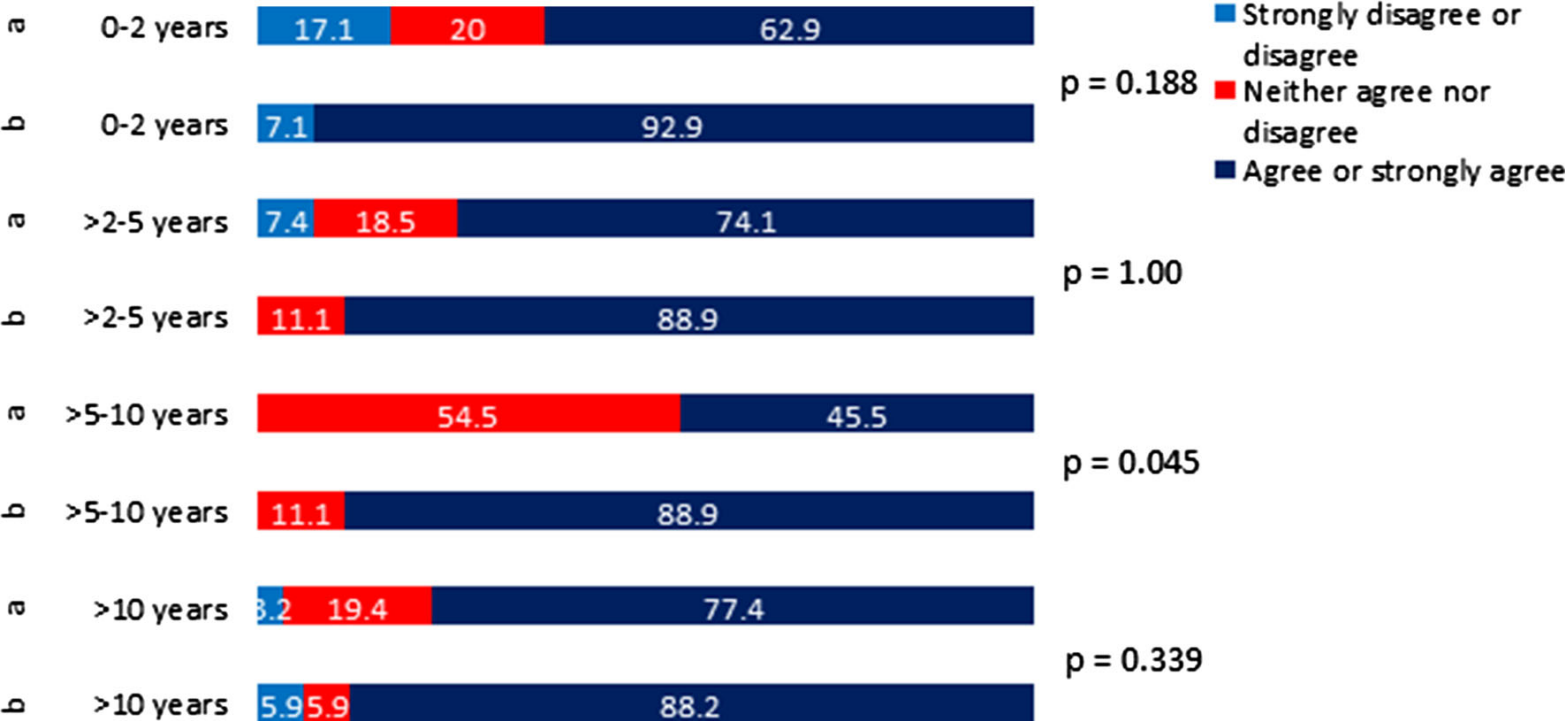
Likert scale answers for *Question 4* **a** directly after the course: “During the scenarios I had the impression that the communication within the team improved”. **b** 3 months after the course: “My way of communicating in emergency situations improved”, plotted against professional experience of participants

Figures 2, 3, 4 and 5 show detailed responses based on level of clinical experience.

Concerning the first question regarding feeling of self-confidence, improvement 3 months after the course did reach statistical significance for all the groups (Fig. 2). For the second question about handling of the emergency situation, the improvement in the self-perceived competency did not reach statistical significance for the group with 5–10 years of professional experience (Fig. 3). For the third question on clinical algorithms, although the overall analysis showed statistically significant improvement in self-perceived competency, only improvement in the group with >10 years professional experience reached statistical significance (Fig. 4). For the question regarding team communication, there was a trend towards improvement after 3 months in the overall analysis; in the group of participants with 5–10 years of professional experience, the improvement was statistically significant (Fig. 5).

## Comment

Our study confirmed that self-perceived competency with higher self-confidency, better handling of emergency situations and remembering the management of algorithm can be improved by simulation training. Strengths of our study include the multi-professional composition of each simulation group, which is more representative of real-life situations on the labour floor, the high number of obstetricians participating, and the high proportion of experienced obstetricians and midwives participating (33.3 % >10 years professional experience), who despite significant obstetrical experience also benefited from simulation training. This result might reflect a high level of motivation and the persistence to achieve a high quality of professional practice for the experienced participants [11].

Reynolds et al. [3] also evaluated the self-perceived impact of simulation training for obstetrical emergencies within a team of a tertiary university hospital and showed an overall improvement in self-perceived competency for 46 professional health care providers 1 year after the course. Compared to that of Reynolds et al. [3], we had a clearly different collective which was composed of significantly more doctors than midwives, from obstetric units with different levels of care and public and private institutions.

In our questionnaire, we could demonstrate a statistically significant improvement in self-perceived competency 3 months after the course compared to directly after the training for the first three questions, but not for the fourth regarding team communication. The participants also gave a low scores for the question regarding training of patient communication. Siassakos et al. [6–8] analysed the component of good team communication and team work in a cross-sectional secondary analysis of the Simulation and Fire-drill Evaluation randomized controlled trial (SaFe Trial) and could identify some team behaviours related to better team performance.

We only evaluated the subjective evolution of performance and, therefore, no conclusions could be made regarding objective improvement in the management of obstetrical emergencies of the individuals and within teams. However, there is increasing evidence that self-confidence is an important mediating factor that contributes to the extent to which one approaches learning and persists towards achievement of goals and expertise [11]. Sorensen et al. [12] showed an improvement of confidence scores after introducing simulation training at their institution, and interestingly, a fewer sick leaves among midwives during the study period. They also found a relationship between level of confidence and scores in a test of clinical knowledge in participants after training.

Siassakos et al. [7] also found no relationship between individual skills and knowledge and team performance. We chose to let the participants with different professional experiences train together in their normal function, postulating that they will benefit most in this way.

Limitations of the study include the low return rate of 36.3 % for the second questionnaire, which is comparable to the return rate described from Gardner et al. [13] (33 %), but significantly lower than in the studies of Reynolds et al. [3] with a return rate of 86 %, or Vadnais et al. [14] with a return rate of 70 and 79 %, in which the participants all belonged to the same institution. Our results cannot be generalized as the study population was selected and the course with its financial costs may have preferentially attracted participants with special interest for this topic. It was organized on a voluntary basis and was not limited to a single institution. The questionnaire was anonymous and we could not determine whether the non-responders of the second questionnaire had different characteristics than the responders. We did not receive information about improvement of clinical skills of the participants.

Therefore, further research should focus on the transfer of improved self-competency to relevant improvement in clinical skill levels, team communication and obstetrical outcomes.
